# Antagonistic Impact of Acrylamide and Ethanol on Biochemical and Morphological Parameters Consistent with Bone Health in Mice

**DOI:** 10.3390/ani10101835

**Published:** 2020-10-09

**Authors:** Monika Martiniakova, Anna Sarocka, Veronika Kovacova, Edyta Kapusta, Zofia Goc, Agnieszka Gren, Grzegorz Formicki, Radoslav Omelka

**Affiliations:** 1 Faculty of Natural Sciences, Constantine the Philosopher University in Nitra, 949 74 Nitra, Slovakia; sarocka.anna@gmail.com (A.S.); vkovacova@ukf.sk (V.K.); 2 Faculty of Exact and Natural Sciences, Pedagogical University of Cracow, 30 084 Cracow, Poland; edyta.kapusta@up.krakow.pl (E.K.); zofia.goc@up.krakow.pl (Z.G.); agnieszka.gren@up.krakow.pl (A.G.); grzegorz.formicki@up.krakow.pl (G.F.)

**Keywords:** acrylamide, alcohol, diet, bone health, biochemical analysis, morphological analysis, microcomputed tomography, mice

## Abstract

**Simple Summary:**

Alcohol consumption, the drinking of beverages containing ethanol, represents a growing problem worldwide. Alcohol intake is often combined with an improper diet based on highly processed starch products that are rich in acrylamide. Both acrylamide and alcohol have a harmful impact on bone health. We previously demonstrated that adverse effects of ethanol on cortical bone structure were partly reduced by a relatively high dose of acrylamide in mice after one remodelling cycle. The present research was designated to reveal whether the antagonistic impact of both aforementioned toxins can also be achieved using a lower dose of acrylamide. According to our results, individual administrations of acrylamide and ethanol had adverse impacts on biochemical and morphological parameters consistent with bone health in mice. However, the most detrimental effects of ethanol were again alleviated by acrylamide at the dose used in this study.

**Abstract:**

The aim of present study was to verify antagonistic effect of acrylamide (AA) and ethanol (Et) on bone quality parameters. Adult mice (*n* = 20) were segregated into four groups following 2 weeks administration of toxins: group E1, which received AA (20 mg/kg body weight daily); group E2, which received 15% Et (1.7 g 100% Et/kg body weight daily); group E12, which received simultaneously both toxins; and a control group. An insignificant impact of individual applications of AA, Et or their simultaneous supplementation on the total body weight of mice and the length and weight of their femoral bones was identified. In group E1, higher levels of alanine aminotransferase (ALT), aspartate aminotransferase (AST), triglyceride (TG), a decreased level of glutathione (GSH) and elevated endocortical bone remodelling were determined. A significantly lower relative volume of cortical bone, bone mineral density (BMD), elevated endocortical bone remodelling and cortical porosity, higher levels of ALT, AST, lower values for total proteins (TP), GSH, alkaline phosphatase (ALP), calcium, and phosphorus were recorded in group E2. In the mice from group E12, the highest endocortical bone remodelling, decreased values for BMD, TP, GSH and ALP and increased levels of ALT and AST were found. Our findings confirmed the antagonistic impact of AA and Et at doses used in this study on biochemical and morphological parameters consistent with bone health in an animal model.

## 1. Introduction

Alcohol beverages containing ethanol (Et) are widely consumed throughout the world. Acrylamide (AA) belongs to the most common toxins in the human diet. It is formed during frying, deep frying and baking foods rich in carbohydrates, and especially in amino acid asparagine [1]. Simultaneous consumption of Et and AA-rich food is widespread among humans. Detrimental effects of AA and Et on various organ systems including the skeleton have been described in several studies [2,3,4,5,6]. Their toxicity contributed to differences in selected biochemical and morphological parameters of various organs. AA is present in all forms of foods prepared at high temperatures, including potato chips, fried potatoes, coffee, cornflakes and bread [1]. It is known that AA is carcinogenic to animals and might pose a risk to human health. According to Benziane et al. [7], peroral exposure to AA (5 mg or 10 mg for 2 months) induces kidney damage, hepatocellular insufficiency, and chronic liver disease, resulting in primary immunodeficiency and activation of the immune system in rats. Excessive alcohol consumption may also cause several pathological conditions, such as liver failure, brain damage, and various form of cancer [8].

With respect to the bone, acute peroral administration of AA (1 mg/kg body weight (bw) in a 24 h and 48 h period) affected the microstructure of cortical and trabecular bone tissues of mice. The cortical bone was more resorbed because of a higher number of resorption lacunae. In the trabecular bone, increased values for relative bone volume and trabecular number were determined [3]. Subacute exposure to AA (40 mg/kg bw for 2 weeks) had adverse effects only on cortical bone microstructure. Acrylamidated mice were shown to have increased levels of alanine aminotransferase (ALT), aspartate aminotransferase (AST), calcium (Ca), and decreased glutathione (GSH) [6]. Subchronic exposure to Et (1.7 g 100%/kg bw for 8 weeks) negatively influenced both the cortical and trabecular bone tissue structures of mice. In the cortical bone, increased porosity and decreased values were established for relative bone volume and bone mineral density (BMD). In the trabecular bone, lower relative bone volume, trabecular number, trabecular thickness, and bone surface were determined [4]. In the study by Broulik et al. [2], femoral bones of alcohol-fed rats (7.6 g of 95% Et/kg bw daily for 3 months) were characterised by a reduction in BMD, mechanical strength, cortical bone thickness, as well as in Ca and phosphate content. The levels of alkaline phosphatase (ALP) and AST did not differ between alcohol-fed rats and control ones. According to Bartlett et al. [9], chronic alcohol consumption induced changes in calcium regulating hormones, mineral homeostasis and mechanical loading and was consistent with an accumulation of reactive oxygen species (ROS) [10]. In addition, osteoblasts of rats injected intragastrically with liquid diets containing Et (12 g Et/kg daily for 4 weeks) had upregulated expression of nicotinamide adenine dinucleotide phosphate (NADPH) oxidase [11].

We previously demonstrated that harmful effects of Et on cortical bone structure were partly reduced by a relatively high dose of AA [6]. The present research was designated to reveal whether antagonistic impact of these toxins can also be achieved using a lower dose of AA after one remodelling cycle.

## 2. Materials and Methods

### 2.1. Animals

The First Local Ethic Committee on Experiments on Animals in Cracow approved all the relevant procedures (resolution number 175/2012). Twenty clinically healthy 12-week-old Swiss mice (males) were used in our experiment. Mice were housed in individual flat-deck wire cages under a 12 h light/dark cycle, a temperature of 20–24 °C and humidity of 55% ± 10% with free access to a standard diet (Agropol, Motycz, Poland) and water on an ad libitum basis. Animals were segregated into 4 groups following 2 weeks of administration of toxins: group E1 received AA (daily dose of 20 mg/kg bw); group E2 received 15% Et (daily dose of 1.7 g 100% Et/kg bw); group E12 was simultaneously supplemented by both toxins (20 mg AA/kg bw + 15% Et) per day; and a control (C) group without AA and/or Et administration per os.

The AA and Et dosages were selected based on studies performed by other authors [6,12,13,14]. Both toxins were dissolved in physiological saline and administered by syringe at established doses perorally to mice. Group C of animals received only physiological saline solution.

### 2.2. Biochemical Analysis

A day after the last toxin application, mice were placed in a state of deep anaesthesia for sacrifice by administration of Vetbutal (35 mg/kg bw; Biowet, Poland), and fasting whole blood samples were taken from the carotid artery. Then, whole blood was processed, and plasma ALP, ALT, AST, total protein (TP), triglyceride (TG), Ca and phosphorus (P) were measured using commercially available kits (Stamar, Dąbrowa Gornicza, Poland). Blood GSH levels were determined by Ellman’s method [15].

### 2.3. Macroscopical Analysis

Bone specimens (both femoral bones) were sampled during necropsy. All femoral bones (*n* = 40) were weighed with a precision of 0.01 g on analytical scales, and their lengths were measured using a sliding instrument. In addition, total body weight of mice from all groups was also determined.

### 2.4. Micro-CT Analysis

Microcomputed tomography (micro-CT) was used to conduct quantitative 3D analysis of cortical and trabecular bone tissues. Within the cortical bone, regions of interest (ROIs), beginning at 5.2 mm from the growth plate at the distal end and extending 1.5 mm in the femoral midshaft, were analysed. The ROIs of trabecular bone started at 1.2 mm from the growth plate at the distal end and continued for 1.5 mm. High-resolution scans were obtained with a voxel size of 6.8 μm. The scanning conditions included 70 kV, 200 μA, 300 ms, 0.5 mm, and an aluminium filter. The micro-CT scans were obtained by μCT 50 (Scanco Medical). The standard analysis programme by Scanco was used to assess specific bone parameters as follows: relative bone volume with and without marrow cavity (%), bone mineral density (BMD; mg HA/ccm), bone surface (mm^2^), cortical bone thickness (mm), trabecular number (1/mm), and trabecular thickness (mm).

### 2.5. Histomorphological Analysis

Histomorphological 2D analysis of cortical bone tissue was performed according to the previously published method [16]. In brief, femoral bones were embedded in epoxy resin, and thin sections were cut with a sawing microtome [17]. Internationally accepted classification systems of Enlow and Brown [18] and Ricqlés et al. [19] were applied to evaluate the qualitative 2D characteristics. The quantitative 2D parameters were calculated using Motic Images Plus 2.0 ML (Motic China Group Co., Ltd.) software. The area (μm^2^) of primary osteons’ vascular canals, Haversian canals and secondary osteons was measured in all views of thin sections in order to minimise statistical differences in the individual.

### 2.6. Statistics

Statistical analysis was performed using IBM SPSS Statistics 26.0 software (IBM, New York, NY, USA). The measured values were expressed as mean ± standard deviation. The differences in investigated parameters among all groups were calculated using analysis of variance (ANOVA) with Games–Howell’s and/or Tukey’s post hoc tests. The compared groups served as an independent variable; the measured parameters were dependent variables. The continuous data had a normal distribution. All *P*-values were considered significant if less than 0.05.

## 3. Results

### 3.1. Biochemical Analysis

**Figure 1:**
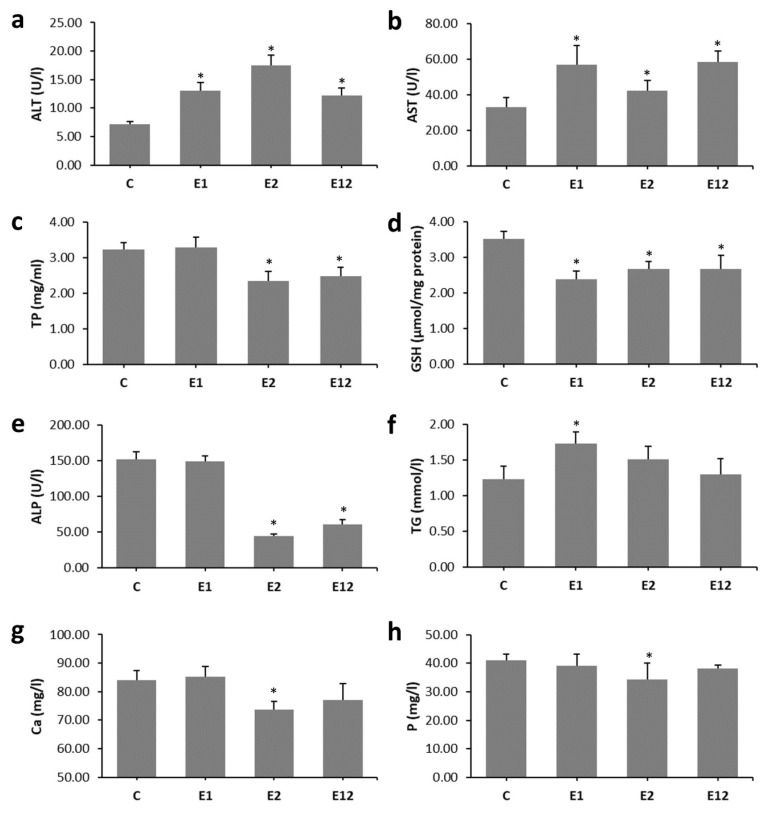
Biochemical parameters consistent with the bone quality in mice from groups C, E1, E2, and E12. (**a**)—alanine aminotransferase, (**b**)—aspartate aminotransferase, (**c**)—total proteins, (**d**)—glutathione, (**e**)—alkaline phosphatase, (**f**)—triglyceride, (**g**)—calcium, (**h**)—phosphorus. * Significant differences in relation to control (*P* < 0.05).

Enhanced levels of ALT, AST, TG and decreased GSH were recorded in group E1 when compared to group C. In mice exposed to Et, higher values for ALT and AST and lower levels of TP, GSH, ALP, Ca and P were determined versus control mice. In group E12, decreased values for TP, GSH and ALP and higher levels of ALT and AST were found as compared to group C (Figure 1a–h).

### 3.2. Macroscopical Analysis

**Figure 2:**
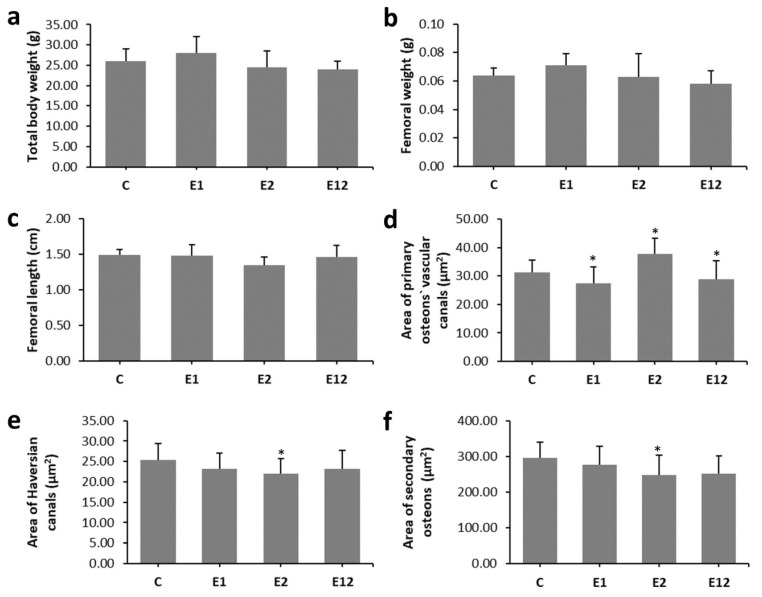
Macroscopical analysis of bones (**a**–**c**) and quantitative 2D analysis of cortical bone tissue (**d**–**f**) in mice from groups C, E1, E2, and E12. * Significant differences in relation to control (*P* < 0.05).

We did not find a significant effect of individual applications of AA, Et or their simultaneous administration on the total body weight of mice or the length and weight of their femoral bones. The results are summarised in Figure 2a–c.

### 3.3. Micro-CT Analysis

**Figure 3:**
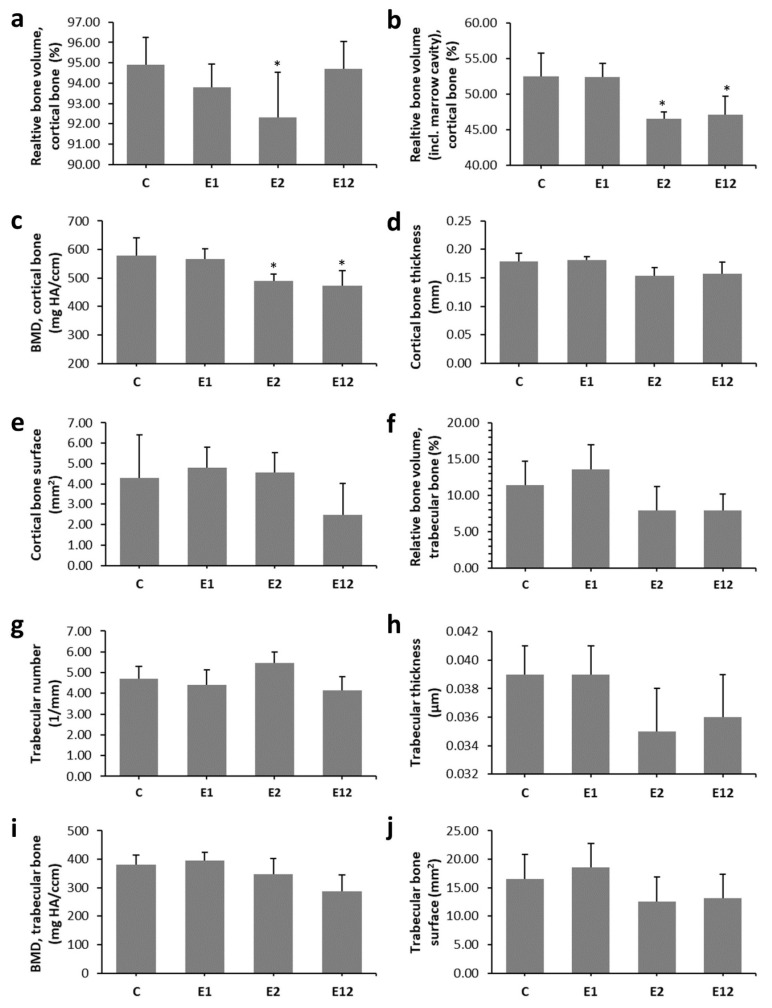
Micro-CT analysis of cortical (**a**–**e**) and trabecular bone tissues (**f**–**j**) in mice from groups C, E1, E2, and E12. * Significant differences in relation to control (*P* < 0.05).

**Figure 4:**
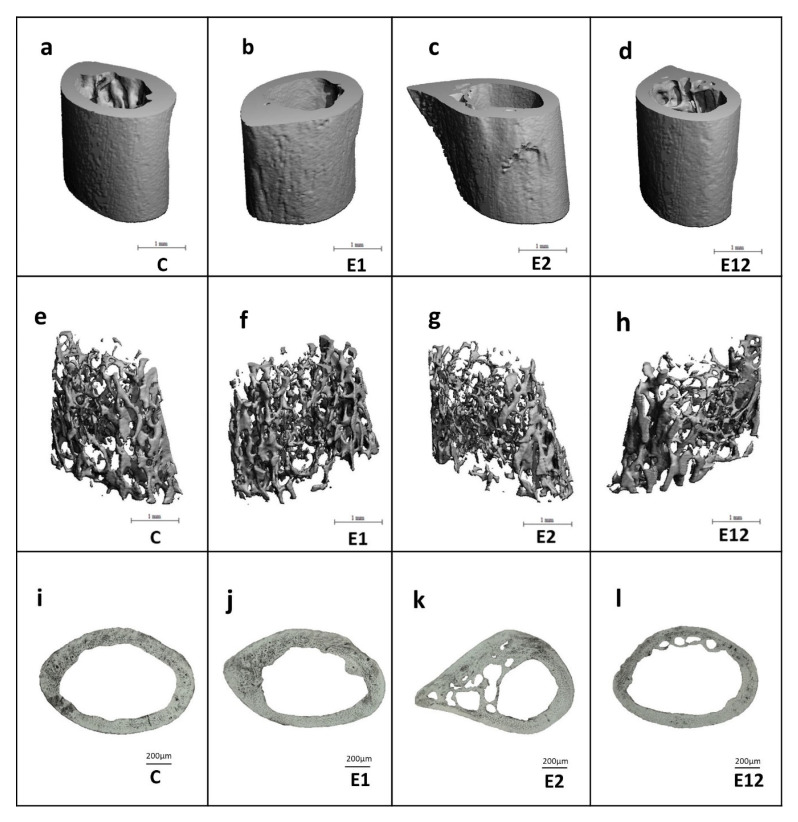
Representative 3D images of cortical (**a**–**d**) and trabecular bone tissues (**e**–**h**) in mice from groups C, E1, E2, and E12. Representative 2D images of cortical bone tissue (**i**–**l**) in mice from groups C, E1, E2, E12.

Micro-CT analysis of the cortical bone showed significantly reduced relative bone volume with and without marrow cavity and BMD in mice from group E2. Lower values for relative bone volume with marrow cavity and BMD were also observed in group E12 versus group C. On the contrary, all measured 3D parameters of the cortical bone were not significantly different between groups E1 and C (Figure 3a–e). Trabecular bone microarchitecture did not change significantly among all groups. The results are documented in Figure 3f–j. Representative 3D images of the cortical and trabecular bone tissues are shown in Figure 4a–d,e–h, respectively.

### 3.4. Histomorphological Analysis

In mice from group C, non-vascular bone tissue formed both surfaces (endosteal and periosteal) of femoral bones. Several secondary osteons were observed in the lateral parts near the endosteum and in the middle parts of the cortical bone. Only medial parts consisted of non-vascular bone tissue. Mice from group E1 had enhanced endocortical remodelling. About 73% more secondary osteons were recorded near endosteal surfaces when compared to group C. Higher density of secondary osteons (about 46%) was also detected near the endosteum in mice from group E2. However, we identified many resorption lacunae consistent with an increased cortical porosity in Et-fed mice. The highest density of secondary osteons of about 140% was determined near endosteal surfaces in group E12. We also recorded several resorption lacune near the endosteum; however, the number of lacune was lower in comparison with group E2 (Figure 4i–l). In total, 757 primary osteons’ vascular canals, 99 Haversian canals and 99 secondary osteons were measured. The area of primary osteon’s vascular canals was significantly decreased in groups E1 and E12 versus group C. On the other hand, mice exposed to Et showed a higher area of primary osteon’s vascular canals. Nevertheless, the area of Haversian canals and secondary osteons was lower in these mice (Figure 2d–f).

## 4. Discussion

Evidence accumulated in recent years has shown that physiological serum levels of liver enzymes (within and just above the normal range) are consistent with an increased risk of incident metabolic diseases. It is also well known that chronic liver disease is associated with profound adverse effects on bone health and homeostasis [20]. Excessive AA and Et consumption can lead to both liver and bone damage.

Our results from biochemical analysis indicate liver disease in all experimental groups of mice exposed to AA and/or Et. The liver failure caused by aforementioned toxins was also revealed in other research and was consistent with enhanced levels of ALT and AST in acrylamidated rats (10 mg AA/kg bw for 21 days) [21] and Et-fed mice (5–6% Et for 10 days to 12 weeks) [22]. Because the liver produces different molecules, it was hypothesised that damage of the liver function will result in osteoporosis by influencing the development of bone-active liver molecules [23]. Even a presence of decreased GSH in all experimental groups could lead to hepatocyte disruption. In groups E2 and E12, the hepatotoxic effect was increased, which could be related to a reduced level of ALP. In the study by Broulik et al. [2], a lower value of ALP was also recorded in rats exposed to Et (7.6 g 95% Et/kg bw for 12 weeks). Harmful hepatocyte changes may also be consistent with lower serum levels of Ca and P in group E2. It is known that acute liver failure is associated with Ca and P imbalance [24,25]. A decreased level of TP in Et-fed mice might also indicate liver disorder. In humans, lower hepatic synthesis of proteins can be a consequence of alcoholism-associated malnutrition [26]. A higher value of TG was found in group E1. Ghorbel et al. [27] also revealed an increased TG level in rats fed with AA. According to Mahmood et al. [28], low doses of AA triggered substantial increases in TG levels and contributed to increased synthesis of plasma lipoproteins and lipid mobilisation from the liver.

According to our results, the total body weight of mice and the length and weight of their femoral bones were not influenced by AA and/or Et supplementation. Similarly, AA caused only intermittent differences in the body weight of B6C3F1 mice receiving AA in the drinking water ad libitum for 2 years (doses of 0.0875, 0.175, 0.35, and 0.70 mM AA) [29]. Furthermore, available epidemiological evidence does not support a completely consistent association between body weight and regular intake of Et. Nevertheless, when Et is consumed with food, Et intake may constitute a risk factor for increased body fat due to passive overconsumption of energy in the form of fat, as well as a decrease in total fat oxidation in the presence of Et [30]. Broulik et al. [2] determined lower final body weight in Et-fed rats versus control ones; however, the difference did not reach statistical significance.

In our study, significantly decreased BMD and relative volume of cortical bone with and without marrow cavity were recorded in Et-fed mice. Similar findings were demonstrated in rats exposed to Et (3, 6, 13, and 35% Et for 4 months [31]; 36% Et for 42 days) and Et-fed mice (10–36% Et for 78 days) [32,33]. In general, decreased rates of bone formation followed by low bone mass and a lower BMD are also seen in alcoholics [26]. Significantly decreased BMD and relative volume of cortical bone with marrow cavity have also been identified in mice in group E12. Interestingly, trabecular bone microarchitecture did not vary significantly among all groups. It is known that the trabecular and cortical bones have different bone remodelling levels. Cortical bone has a large volume of matrix and a limited surface area; hence, signals deep within the matrix may not locate a surface as lightly to initiate remodelling, allowing for the microdamage accumulation mostly in less remodelled interstitial bone [34]. Generally, trabecular bone is remodelled more vigorously. However, the surface-to-volume ratio is much greater [35] and the length of one remodelling cycle in the trabecular bone is longer than in the cortical bone.

Mice from groups E1, E2 and E12 demonstrated enhanced intracortical bone remodelling. In general, bone remodelling is the principal mechanism for maintaining a healthy skeleton in adults, and dysfunction in bone remodelling may contribute to bone loss and/or lower bone quality [36]. According to Piemontese et al. [37], intracortical remodelling is associated with increased cortical porosity and formation of secondary osteons that exhibit histologic hallmarks of remodelling activity in mice. Increased intracortical remodelling was also recorded in our previous researches in mice subacutely exposed to AA [6] and in Et-fed mice after 8 weeks [4].

Our findings showed vasoconstriction of primary osteon’s vascular canals in groups E1 and E12. Blood vessels located in vascular canals provide nutrients for the bone [38] and, in response to continuous functional changes, can modify its structure (vascular remodelling) [39]. Generally, AA reduces the high-density lipoprotein (HDL) [40]. Low HDL is related to arterial and vessel narrowing or blockage [41]. On the other hand, Et has a significant influence on the cardiovascular system, such as peripheral vasodilation [42], which can be consistent with increased sizes of primary osteon’s vascular canals in group E2. On the contrary, the area of Haversian canals and secondary osteons was significantly lower in Et-fed mice. Secondary osteons, as products of bone remodelling, are composed of hydroxyapatite crystals and collagen fibres around the central Haversian canal. According to Romero et al. [5], Et consumption reduces the density of mature collagen fibres. Therefore, lower sizes of secondary osteons could be connected with decreased bone toughness [43].

## 5. Conclusions

This study clearly demonstrates that individual administrations of AA (20 mg/kg bw) and Et (15%) had detrimental effects on biochemical and morphological parameters consistent with bone quality in mice. However, the most adverse impacts of Et were alleviated by AA. Although the dose of AA used in this study had fewer negative effects on cortical bone structure compared to the previously examined relatively high dose (40 mg/kg bw), the antagonistic impact of AA and Et on the bone health parameters was again confirmed. We can conclude that the harmful effects of Et on bone quality can be effectively reduced by AA even at lower dose. Simultaneous consumption of Et and AA-rich food is therefore less bad for bone health than individual Et intake.
